# Plasmon-enhanced reduced graphene oxide photodetector with monometallic of Au and Ag nanoparticles at VIS–NIR region

**DOI:** 10.1038/s41598-021-99189-w

**Published:** 2021-10-04

**Authors:** Nurul Syazwani Rohizat, Atiena Husna Abdullah Ripain, Chin Seong Lim, Chee Leong Tan, Rozalina Zakaria

**Affiliations:** 1 Photonics Research Centre, Faculty Science, Universiti Malaya, 50603 Kuala Lumpur, Malaysia; 2 Department of Mechanical, Materials and Manufacturing Engineering, University of Nottingham Malaysia, Jalan Broga, 43500 Semenyih, Selangor Darul Ehsan Malaysia; 3 School of Electronic and Optical Engineering and College of Microelectronics, Nanjing University of Posts and Telecommunications, Nanjing, 210023 China

**Keywords:** Optical properties and devices, Nanoparticles

## Abstract

Hybrids plasmonic nanoparticles (NPs) and unique 2D graphene significantly enhanced the photoresponse of the photodetectors. The metallic NPs that exhibit localized surface plasmon resonance (LSPR) improves strong light absorption, scattering and localized electromagnetic field by the incident photons depending on the optimum condition of NPs. We report high-performance photodetectors based on reduced graphene oxide (rGO) integrated with monometallic of Au and Ag nanoparticles via a familiar fabrication technique using an electron beam evaporation machine. Under 680 nm illumination of light, our rGO photodetector exhibited the highest performance for Au-rGO with the highest responsivity of 67.46 AW^−1^ and the highest specific detectivity (2.39 × 10^13^ Jones). Meanwhile, Ag-rGO achieved the highest responsivity of 17.23 AW^−1^, specific detectivity (7.17 × 10^11^ Jones) at 785 nm. The response time are 0.146 µs and 0.135 µs for Au-rGO and Ag-rGO respectively for both wavelengths. The proposed photodetector with combining monometallic and graphene provide a new strategy to construct reliable and next-generation optoelectronic devices at VIS–NIR region.

## Introduction

2D graphene is a promising candidate material to be applied in broadband photodetectors as it has strong light-matter interaction. The ability to detect light in this region has been widely utilized in various applications, including free-space communications, surveillance, chemical sensing and biomedical imaging^1^. The optical absorption coefficient of single-layer graphene is at $-\mathrm{ln}\frac{\left(1-\pi \alpha \right)}{d}\approx 7 \times {10}^{5} {\mathrm{cm}}^{-1}$, independent on the wavelength where *d* = 0.355 nm (thickness of graphene and $\propto$ = constant of structure^2^. Graphene oxide (GO) is a promising material and beneficial for graphene-based applications like sensor, gas transport, water treatment and energy storage where weak optical absorption is adequate^3^. This is due to short interaction length of a graphene layer where graphene only absorbs $\pi \alpha =2.3\%$ on incident light^2^. As a single layer of carbon atoms, graphene exhibits strong light-matter interaction with photons where it is most desired in optoelectronics and nanophotonic devices where graphene itself tight with weak absorption^4^. There are many approaches to enhance the optical absorption for these applications. The effect of GO reduction process on the photoresponse capability is reported by Carmela Bonavolontà et al.^5^ revealed broad absorption range of the GO and rGO thin films, where rGO/Gr demonstrates a photoresponse at a broad spectral range with a maximum responsivity and detectivity of 0.20 AW^−1^ and 7 × 10^10^ cmHzW^−1^, respectively. One possible way of overcoming these weakness is to utilize plasmonic nanostructures where incident light absorbed by such nanostructures, can be efficiently converted into plasmonic oscillations like Au-ZnO^6^ and Zn–S on rGO^7^ GaN-graphene-Si heterostructures^8^ which leads to a dramatic enhancement of the local electric field. Echtermeyer et al.^9^ reported the efficiency of graphene-based photodetectors increased up to 20 times shows the effectiveness in comparison with III–V material. Abid et al.^10^ reported highest sensitivity of 49.2% is obtained at 123 K for 635 nm laser at power density of 1.4 mW mm^−2^ using rGO photodetector.

In the beginning, GO is only a precursor material for a low-cost and simple method to prepare and produce single layer and multilayer graphene by reduction. Further studies show that the substantial structure imperfection of graphene oxide derived materials due to defects in initial graphite and incompletion of reducing process. GO has the same hexagonal carbon structure to graphene. It also contains hydroxyl (–OH), alkoxy (C–O–C), carbonyl (C=O), carboxylic acid (–COOH) and other oxygen-based functional groups^11^. More importantly, GO can be utilized to synthesis reduced graphene oxide (rGO) by several steps of procedures. It is known that the reduction of graphene oxide yields rGO. This is to reduce the number oxygen groups and attain properties near-pristine graphene^3^. The reduction can be achieved by several methods; thermal, chemical and electrochemical. In this study, thermal method is being applied for its simplicity. With regards to this reduction method, conductivity of rGO can be tuned by tuning the annealing temperature and the number of oxygen atoms^12^.

Noble metal nanoparticles exhibit novel properties that significantly different from those of corresponding bulk-size due to their small size and large surface/volume ratio and are intensively studied due to their excellent properties and applications^13^. Many studies have been done using metallic nanoparticles for their wide range of applications namely in the field of optoelectronic, catalysis, medical applications, energy-based research, sensors and diagnosis. For particles below 100 nm in diameter, it is considered as nanoparticles as this is where the properties are different from its bulk-size form. Size, shape and structure of these metallic nanoparticles are known to play important role from their properties^14^. Metallic nanoparticles incorporated with graphene has emerged as an effective method to enhance the light-matter interaction for application in optoelectronic devices due to their unique plasmon properties called localised surface plasmon resonance (LSPR)^15–17^. The incorporation of noble metallic nanoparticles in graphene may produce plasmonic effects that lead to the enhancement of optical absorption, resulting in improved performance of optoelectronic devices.

This research investigates the different properties between the monometallic of silver (Ag) and gold (Au) nanoparticles using the familiar fabrication technique of an electron-beam evaporation machine. The electron beam dose effects on sintering of passivated Au and Ag nanoparticles driven by surface atom diffusion rather than Ostwald ripening^18^. The transformation of a thin film to a favoured set of droplets and particles are called dewetting where it denominates the process to occur below the melting temperature. Applications of the produce nanoparticles are envisaged to range from optical sensors and plasmonic system^19^ since many years ago. The preferences and choice of metallic always refers to response curve of SPR which is very sensitive to real and imaginary part of *ε*_r_ and *ε*_i_, because of they account for reflection and absorption of light in the metal, respectively. Narrow resonance is obtained in the SPR reflection spectra due to small damping if |ε_r_|»1 and |ε_r_|»|ε_i_|^20,21^. This means that the sharpest peak is produced by the metal whose dielectric constants have the highest |ε_r_/ε_i_| ratio where Ag (|ε_r_/ε_i_|= 38.0) is indeed the case ^3,5,6^. Gold (|ε_r_/ε_i_|= 7.33) produces a broader peak than Ag, however, it also gives good SPR spectra due to the inertness^22^ as well as integrating these particles proved an efficient way in various distinctives features like light trapping, high efficiency, strong absorption, and improved sensitivity^23^. Here, we show that the reduced graphene oxide incorporated with plasmonics nanoparticles as photodetectors extremely enhance strong-light matter interaction and thus improve the performance of the sensor device.

## Experimental section

### Fabrication of nanoparticles

A thin layer of metallic deposited on a silicon substrate homogenously using electron beam evaporation machine. In this study, monometallic nanoparticles were synthesized on Si and glass substrate. Glass substrate was used as a reference. Both substrates were cleaned with (in the order of) acetone, iso-propanol solutions and distilled water for 5 min each. All samples are then dried using nitrogen gas. The Au metal films were first deposited on the substrates using e-beam evaporation method under a pressure of 1 × 10^–6^ Torr and at a low deposition rate of 0.2–0.5 Å/s to improve the uniformity over a large surface area. Au and Ag metal were chosen because of the two different LSPR wavelengths (around 600 and 400 nm, respectively) that are significantly apart and because the difference in the melting temperatures for the two metals is substantial^24^.

To form the nanoparticles, thermal dewetting process was used. The Au thin metal film was first annealed at a temperature T_1_ = 600 °C for 1 min. The thin metal film changes into either hemisphere-shaped NPs or a metal cluster which according to Mueller et al.^25^, if the temperature is increased up to critical temperature, both structures still maintain the same shape, beyond which the particles melt and evaporate. Ag nanoparticles layer produced from a silver thin film when annealed at 250 °C. The formation of nanoparticles from both Au and Ag thin film was due to the reduction of the surface energy during elevated temperature which highly dependent on the metallic properties. This method is a preferable as it results in highly reproducible and uniform surface^26^.

### Device fabrication

**Figure 1:**
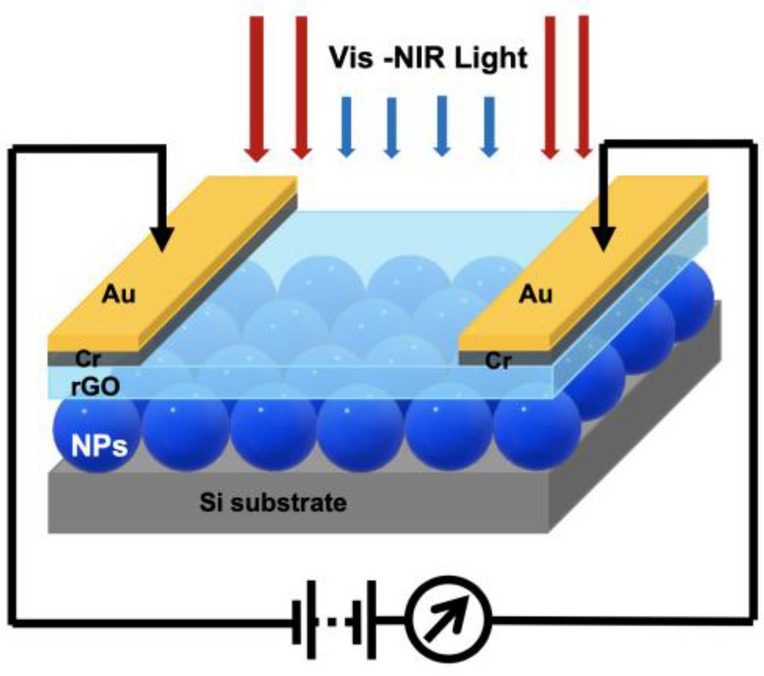
Schematic of plasmonic metallic NPs based on the rGO-Si photodetector.

The photodetector devices were prepared by first fabricating the metal nanostructures on Si substrates followed by deposition of Cr/Au electrodes at ~ 1 Å deposition rate under a pressure of 1 × 10^–6^ Torr, respectively. The thickness of the Cr layer was 5 nm and the thickness of Au layer deposited was 80 nm. Graphene oxide (GO) is bought from Graphenea with concentration of 4 mg/ml. GO is then mixed with a solvent, namely ethanol and is diluted to ratio of 1:15. By mixing GO with ethanol could improve its dispersion^27^. The GO solution with ethanol was sonicated for 2 h to form a stable and uniform GO solution. The new diluted GO solution is deposited on the silicon-based substrate with NPs and electrodes via spray deposition with controlled volume of 10 ml each time. The device was then placed on a hotplate (180 °C) to facilitate solvent evaporation. This step is also crucial for the reduction of GO to rGO. The fabricated devices have same effective contact area as 1 × 1.5 cm^2^, namely the area of p-Si covered by rGO film. Figure 1 shows the diagram structure of the rGO-Si photodetector.

## Results and discussion

### Characterization of Au and Ag NPs

**Figure 2:**
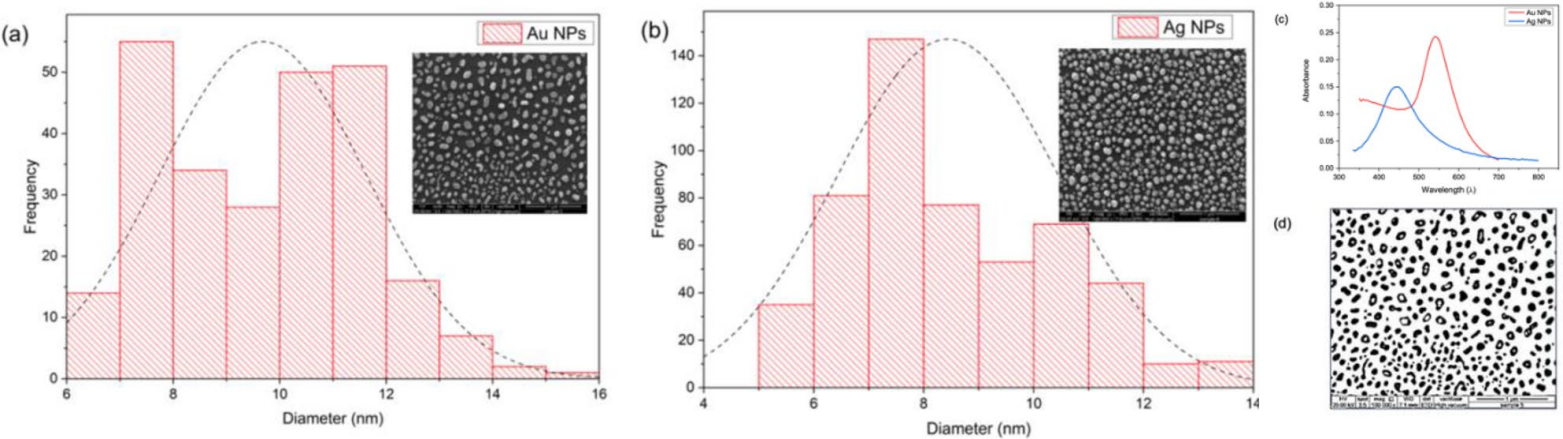
The SEM images and their respective histograms showing size of the nanoparticles with inset images as (**a**) Au NPs (**b**) Ag NPs (**c**) UV–Vis absorption spectra and (**d**) image-J particles calculation.

The images of all two types of monometallic nanoparticles were observed using Field Effect Scanning Electron Microscopy (FESEM). The displayed FESEM images are nanostructures derived from the thermal dewetting process. After annealing at specific temperatures and duration, the isolated and evenly distributed Au and Ag NPs were formed as displayed in inset image in Fig. 2. Their corresponding histogram graph shows in Fig. 2a,b. The histogram refers to the size distribution of the nanoparticles plotted from the images which taken at magnification of 100,000×. These images of Au and Ag NPs depicts that the nanoparticles are monodispersed and not aggregated. The Ag NPs have spherical in shape while Au NPs is slightly elongated. Most of the nanoparticles’ size are in the range of ~ 7 nm to 10 nm in average obtained from histograms. The UV–Vis absorption spectra are shown in Fig. 2c. As displayed in the Fig. 2c, the distinctive peak of Au NPs is 550 nm and Ag NPs is blue-shifted at 440 nm. All the particles size calculated using the open-source microscopic image processing software, ImageJ by calculating the diameter (*d*) in pixels illustrated in Fig. 2d.

### Electrical characterizations of monometallic Au and Ag NPs

All electrical measurements were carried out under ambient conditions and the dark currents were measured in a dark box to eliminate the interference of ambient light interference. The optoelectronics performance of photodetectors was measured using Keithly source meter (2410) with potential scanning from − 30 to 30 V at different light power intensities. The photoresponse (I–t) measurements of the devices were characterized under zero bias and potential by Yokogawa DLM2054 oscilloscope and Stanford Research synthesized function generator (Model DS345). The LED light sources are 680 nm and 785 nm were applied as the illumination light source with adjustable light power intensity from 0.01 to 1.11 mW cm^−2^ (680 nm) and 0.03 to 15.08 mW cm^−2^ (785 nm). They are calibrated by THORLABS optical power meter.

**Figure 3:**
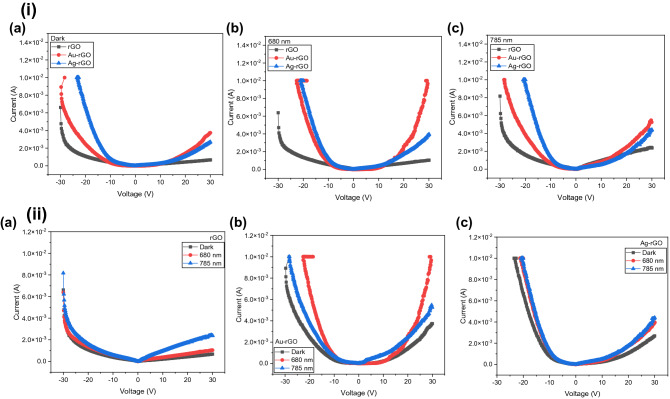
Current–voltage (I–V) curves (i) (**a**) by condition as at dark, (**b**) 680 nm and (**c**) 785 nm illumination and (ii) by device comparison as (**a**) bare-rGO, (**b**) Au-rGO and (**c**) Ag-rGO.

The current–voltage (I–V) was conducted with fixed 100% light intensity of 1.11 mW cm^−2^ and 15.08 mW cm^−2^ respectively. The voltage range was varied between ± 30 V in the dark and under illuminations. A significant difference of the dark and photocurrent were displayed in the devices both with and without NPs as shown in Fig. 3. Total current is deducted with dark current to obtained the net photocurrent I_p_ of light illuminated. Under dark, an increase of current was observed in the order of 10^–4^ (bare rGO) to 10^–3^ A (Au- and Ag NPs) at 30 V as shown in Fig. 3(i)a–c. This depicting the rectifying behaviour which indicate the formation of Schottky type barrier in the devices. This is due to layer of rGO absorbs incident light, where excitons (electron–hole pairs) are obtained at the Schottky-like metal-rGO interface. In addition, defects in the rGO film can help dissociate excitons into free carriers and some of them have sufficient energy to overcome the Schottky barrier. It was observed in Fig. 3(ii)a–c shows the trend comparison for all conditions of the device, where the I–V curves was sharply increase with the presence of Au and Ag NPs. In this device, surface plasmons enhance the photocurrent in two ways: by transferring hot electrons generated from plasmon decay in the metal structure and by enhancing the near-field and direct electron–hole pair generation in graphene and contribute to a higher responsivity^28^. This shows a similar trend reported as a photodetector based on hot electron injection into graphene shows nanoscale plasmonic antennas using gold heptamer are sandwiched between two graphene monolayers yield a photodetector that efficiently converts visible and near-infrared photons into electrons with enhancement up to 800% in comparison with bare-graphene photodetector^29^.

**Figure 4:**
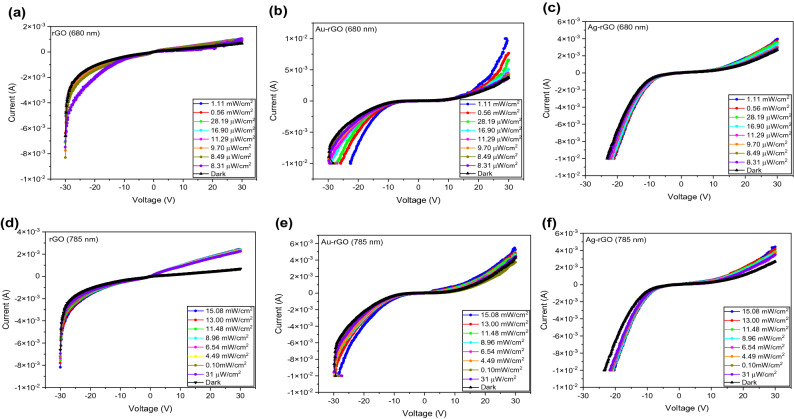
I–V characteristics of bare rGO, Au-rGO and Ag-rGO PD at different power between (**a**–**c**) 8.31 µW cm^−2^ to 1.11 mW cm^−2^ under laser irradiation of 680 nm. (**d**–**f**): 31 µW cm^−2^ to 15.08 mW cm^−2^ under laser irradiation of 785 nm.

With the illumination of visible and NIR light, the photocurrent of bare rGO, Au and Ag was increased throughout the voltage range. The photoexcitation of all the devices were further studied by illuminated with different light intensity. The 680 nm light source is extending from 8.31 µW cm^−2^ to 1.11 mW cm^−2^ as display in Fig. 4a–c while as shown in Fig. 4d–f, the light intensity for 785 nm light source is ranging from 31 µW cm^−2^ to 15.08 mW cm^−2^. Taken together, these results indicated that the current is dependent on light intensity. The graphs display that when light intensity increases, the current increases as well.

**Figure 5:**
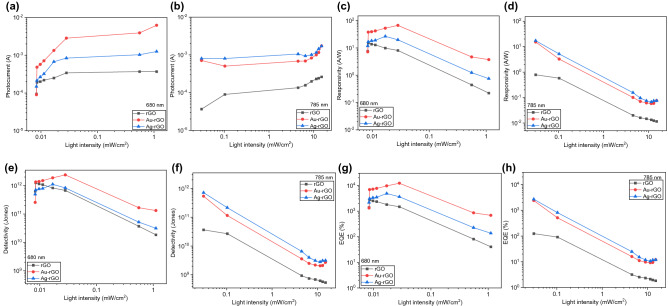
(**a**,**b**). Plot of net photocurrent versus light intensity under laser illumination at 680 nm and 785 nm. (**c**,**d**) The power-dependent responsivity. (**e**,**f**) The light density-dependent detectivity of (**g**,**h**) External quantum efficiency, EQE versus optical power density of the devices.

The performance of the device was further appraised by ascertaining the parameters of the figure of merit (FOM) for instance, responsivity (*R*), specific detectivity (*D*) and external quantum efficiency (EQE) at different power. The responsivity (*R*) and specific detectivity, (D*) are the essential parameters for performance evaluation of the PDs. The net photocurrent, I_p_ of the devices is obtained by deducting dark current from total current (Fig. 5a,b). It can be simplified as in Eq. (1):

1 $${I}_{ph}= {I}_{light}- {I}_{dark}$$

where *I*_*dark*_ is the dark current and *I*_*light*_ is the current under illumination. The responsivity, *R* denotes the device sensitivity of incident light and is describes as the ratio of photocurrent generated to illuminated optical power intensity and calculated as Eq. (2):

2 $$R=\frac{{I}_{ph}}{{P}_{opt} \cdot A}$$

where I_ph_ is the photocurrent, P_opt_ is the power density of incident light (in mW cm^−2^) and A is the total effective illuminated area (in cm^2^) of the device. The results obtained from the calculation of responsivity of all devices can be compared in Fig. 5 for (c) 680 nm and (d) 785 nm respectively. *R* calculated as 8.31 × 10^−3^ mW cm^−2^ and 3.1 × 10^−2^ mW cm^−2^ at 680 nm and 785 nm respectively. Responsivity value shows maximum at lower power and exponentially decreased with increasing power. It is definitely the case for 785 nm, however, at 680 nm laser irradiation, maximum R is calculated to be in between optical power of 8.50 × 10^−3^ to 2.82 × 10^−2^ mW cm^−2^. This is due to the minimum light illumination power is already low and the difference between the optical power itself is too small, hence giving no much changes in reading which is less than 1.13 × 10^−2^ mW cm^−2^ each.

The calculated data found that the responsivity value was significantly enhanced with the integration of Au and Ag nanoparticles. For 680 nm, photodetector with Au NPs shows high responsivity of 67.46 AW^−1^ and Ag NPs is 26.73 AW^−1^. These two devices increase about 4.47 and 1.77 times higher than bare rGO which is 15.09 AW^−1^. Similarly, 785 nm laser irradiation responsivity also exhibits an increment with the incorporation of Au and Ag NPs at 15.29 AW^−1^ and 17.23 AW^−1^, about 19.41 and 21.88 times higher than bare rGO. Responsivity of bare rGO calculated as 0.79 AW^−1^. Moreover, another important variable is detectivity (D*) which implies the capability of a PD to detect a very weak optical signal and is expressed by the Eq. (3):

3 $${D}^{*}=\frac{R \cdot \sqrt{A}}{\sqrt{2q{I}_{d}}}$$

where, *R* is a responsivity, q is electron charge and I_d_ is the dark current of the device. It is noted that when the D* is higher, it is preferable in detecting weak signals. Likewise, D* is higher with the incorporation of Au and Ag NPs in the photodetectors as shown in Fig. 5e,f. Au NPs photodetector displays high value of 2.39 × 10^12^ Jones, which is 2.15 and 1.90 times higher than Ag NPs and bare GO-rGO photodetectors under 680 nm laser light. Irradiation of 785 nm laser yields high detectivity with Ag NPs photodetector at 7.17 × 10^11^ Jones which is just 1.33 times higher than Au NPs and a big gap of 19.64 times greater than bare rGO photodetector.

The maximum responsivity (*R*) and detectivity (D*) of 680 nm illuminations are found to be ~ 67.46 AW^−1^ and 2.39 × 10^12^ Jones at low light illumination power density of 28.19 µW cm^−2^ owned by integration of NPs. At 31 µW cm^−2^, for 785 nm illumination, responsivity (*R*) and detectivity (D*) are ~ 15.29 AW^−1^ and 5.41 × 10^11^ Jones.

Another critical parameter for PD is external quantum efficiency (EQE), calculates the ratio of photocurrent to the number of incident photons by employing the responsivity value. It is represented as Eq. (4):

4 $$EQE=R \times \frac{1240}{\lambda } \times 100\%$$

where λ is excitation wavelength (680 nm and 785 nm). The EQE of rGO, Au-rGO, Ag-rGO photodetectors likewise displayed same trend for 680 nm and 785 nm peak wavelength.

Figure 5g depicts the effective value of the Au NPs incorporated rGO photodetector exhibited maximum EQE of 1.23 × 10^4^% at 680 nm illumination are 2.52 and 4.47 times higher than Ag NPs and bare rGO photodetectors with the EQE of 4.87 × 10^3^% and 2.75 × 10^3^%. At peak wavelength of 785 nm as shown in Fig. 5h, Ag NPs photodetector displays highest EQE of 2.72 × 10^3^%, 1.13 and 21.88 times higher than Au NPs and bare GO-rGO PDs with the EQE of 2.41 × 10^3^% and 1.24 × 10^2^%.

EQE was high at low power density, i.e. 8.31 × 10^–3^ mW cm^−2^ (680 nm) and 3.1 × 10^–2^ mW cm^−2^ (785 nm) and steadily decreased with the increased of power as demonstrated in Fig. 5. Summary of all values are shown in Table 1. As this value of EQE were higher than those previously reported in reference^29,33^ tabulated in Table 2.

**Table 1:** Summary of the figure of merit of all three devices; Au NPs-rGO, Ag NPs-rGO and rGO under wavelength of (a) 680 nm and (b) 785 nm.

Device	Responsivity (A/W)	Detectivity (Jones)	EQE (%)	Rise time (µs)	Fall time (µs)
**(a) 680 nm**					
Au-rGO	67.46	2.39 × 10^12^	1.23 × 10^4^	0.15	0.08
Ag-rGO	19.94	8.3 × 10^11^	3.64 × 10^3^	0.18	0.10
rGO	8.12	6.76 × 10^11^	1.48 × 10^3^	0.85	0.02
**(b) 785 nm**					
Au-rGO	15.29	5.41 × 10^11^	2.41 × 10^3^	0.17	0.11
Ag-rGO	17.23	7.17 × 10^11^	2.72 × 10^3^	0.14	0.09
rGO	0.79	3.65 × 10^10^	1.24 × 10^2^	0.50	0.02

**Table 2:** Comparison of this work with earlier reported rGO photodetector.

Interface	Wavelength	Responsivity (A/W)	Detectivity (Jones)	EQE (%)	Τ_rise_/τ_fall_	References
rGO/AuNPs/Si	532 nm	10.05	–	–	–	^35^
p-rGO/n-Si	600 nm	1.52	–	–	2.0 ms/3.7 ms	^36^
n-rGO/p^+^-Si	830 nm	16.7	2.56 × 10^12^	2.50 × 10^3^	460 µs/446 µs	^37^
AgNPs/rGO/SiO_2_/Si	410 nm	2.03 × 10^–4^	–	–	–	^38^
Ag/rGO/Ag/quartz	632 nm	0.23	–	88	–	^39^
AuNPs-rGO/SiO_2_/Si	White light	3.8 × 10^–2^	2.12 × 10^8^	–	393 ns/399 ns	^40^
Ag NPs/p-NiO/n-rGO/ITO	365 nm	7.2 × 10^–2^	3.95 × 10^12^	24.46	0.80 s/0.84 s	^41^
TiO_2_/rGO/SiO_2_ /Si	370 nm	7.71	7.92 × 10^13^	–	43 ms	^42^
rGO/AuNPs/Si	680 nm	67.46	2.39 × 10^12^	1.23 × 10^4^	0.146 µs/0.082 µs	This work
rGO/AgNPs/Si	785 nm	17.23	7.17 × 10^11^	2.72 × 10^3^	0.135 µs/0.098 µs	

EQE values are found to be more than 100% in this work as much reported by Zhao et al. in polymer photodetector application^30^. Generally, the improvement in EQE occurs because of photons are absorbed by the metallic NPs and represents the effect of more or less carrier generation and recombination. These mechanisms can be simplified as; junction between Au/Ag NPs and rGO/Si able to facilitates the separation of excitons and the extraction of carriers, the surface of Au/Ag NPs are possibly trap minority carriers or depress the carrier recombination in rGO, hot carriers generated in Au/Ag NPs are able to be transferred into graphene thus increases the photocurrent effects and also the near-field enhancement in the surface of Au/Ag NPs are due to the plasmonic effect increases light harvesting in graphene^31^.

**Figure 6:**
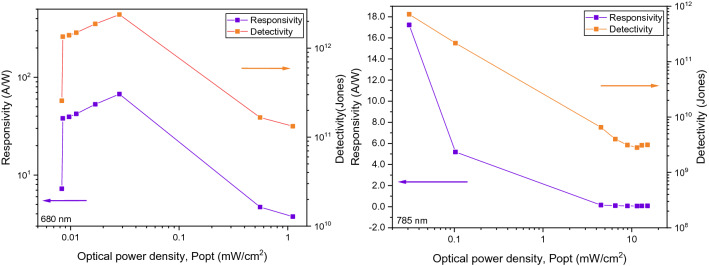
Light intensity-dependent responsivity and detectivity of Au NPs photodetector under illuminations of 680 nm and 785 nm.

In Fig. 6, the correlation data of the responsivity, *R* and the specific detectivity, D* of the Au NPs-rGO PD are presented at 680 nm and 785 nm. From the data in the Fig. 6, it is apparent that the variation *R* and D* with ranging incident power densities contain of two regions. The first region is a linear dependence at low power densities whereas the second region showcase a sublinear behaviour at high power densities. The detected values of *R* and D* at lowest power density implies the ultrasensitive of this PD at both light illuminations^32^.

A test circuit connecting the device, function generator and oscilloscope was performed to further study the response of the photodetector. The function generator is used to modulate the frequency of the light source while the oscilloscope measures and displays the photocurrent as a function of time. The photon-power dependent photoresponse of each device was evaluated at fixed bias 10 V. The photocurrent is plotted as a function of time with different light intensities on the photodetector. A continuous five on–off switching cycles show the rise and decay of photocurrent, maintaining a uniform trend for each cycle.

**Figure 7:**
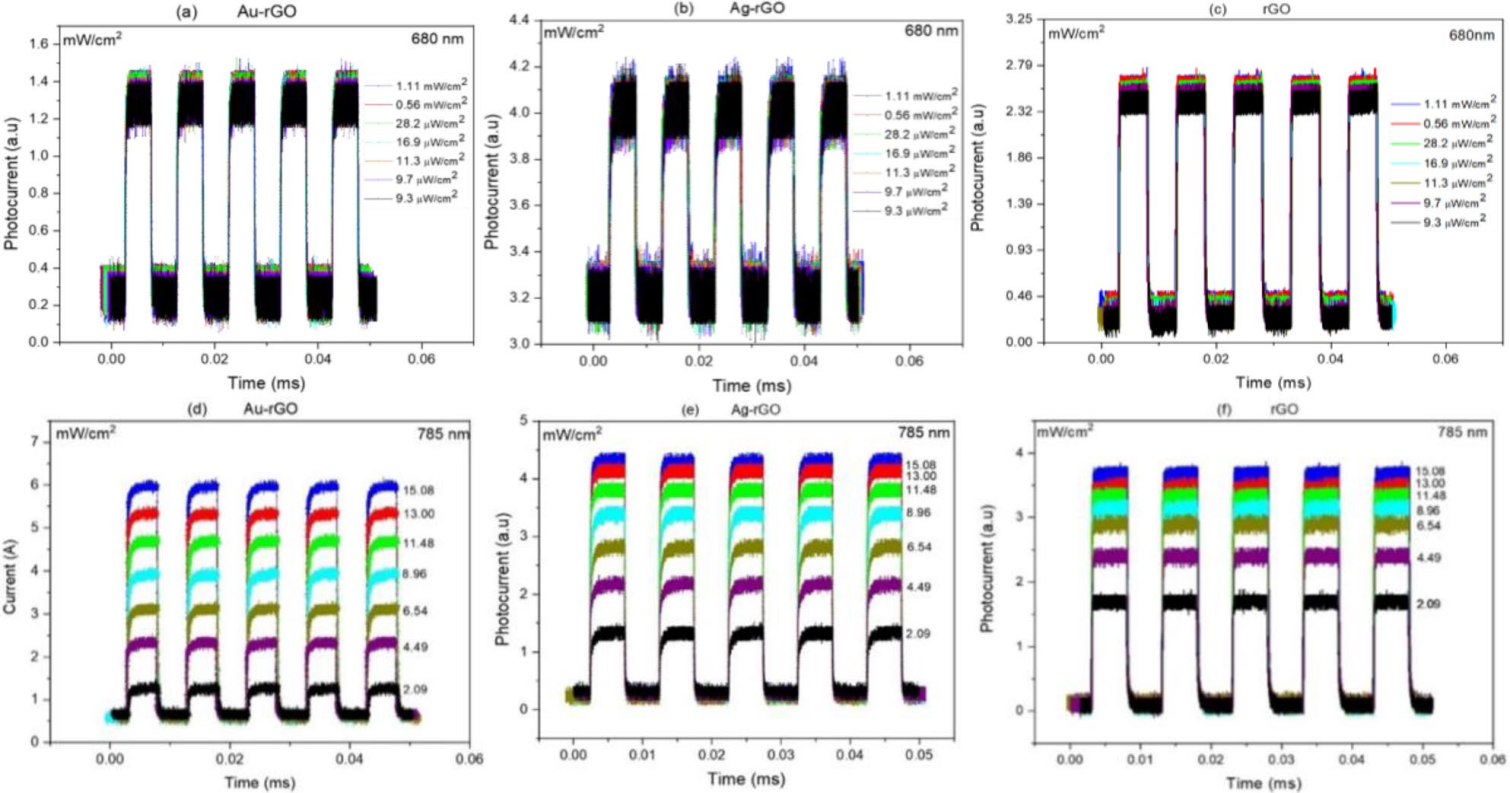
Photoresponse of all devices; Au-rGO, Ag-rGO & rGO PDs illuminated by (**a**–**c**) 680 nm laser and (**d**–**f**) 785 nm at different laser powers.

As shown in Fig. 7a–c, all three devices show a consistent enhancement in the photocurrent when illuminated with LED light of 785 nm wavelength with the power variation between 2.09 and 15.08 mW cm^−2^. However, when illuminated with 680 nm displayed in Fig. 7d–f with power variation between 9.3 µW cm^−2^ and 1.11 mW cm^−2^, no distinct increment was observed. This may be because of the variation of power that is too small to show any significant increment.

**Figure 8:**
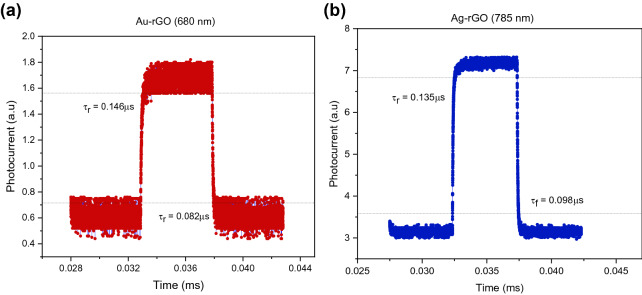
Rising and falling edges for estimating rise time (τ_r_) and the fall time (τ_f_) of (**a**) Au-rGO under 680 nm laser illumination and (**b**) Ag-rGO under 785 nm laser illumination. Both devices show best response time at particular wavelength.

There are two fundamental properties of the photoresponse involves speed, rise time, ${\tau }_{r}$ and fall time, ${\tau }_{f}$ as shown in Fig. 8. The term rise time denote the time required to rise from 10 to 90% of maximum current while the term fall time is described as the time it takes to decrease from 90 to 10% of falling edge, respectively. At peak wavelength of 680 nm, the Au-rGO demonstrate shorter response time, ${\tau }_{r}{:}$ 0.15 µs and ${\tau }_{f}{:}$ 0.08 µs. The Ag-rGO PD on the other hand displayed a bit longer of response time, ${\tau }_{r}{:}$ 0.18 µs and ${\tau }_{f}{:}$ 0.10 µs. This may be because of lifetime of Ag NPs on the Fermi surface is prolonged than Au NPs^33^. And it is vice versa at 785 nm, similar as FOM parameters which show Ag NPs-integrated PD is better than Au NPs-incorporated at 785 nm illumination, i.e. ${\tau }_{r}{:}$ 0.14 µs and ${\tau }_{f}{:}$ 0.09 µs (Ag NPs PD), ${\tau }_{r}{:}$ 0.17 µs and ${\tau }_{f}{:}$ 0.11 µs (Au NPs PD). Huang Fan et al.^34^ produced all type of devices with narrow distribution for photocurrent and response time, suggesting that semiconductor film with fixed amount of photo-generated carriers and uniform charge transfer behaviour at semiconductor-graphene interface. This indicates the potential for standardizing the production of high- and reproducible-performance graphene/semiconductor film hybrid photodetectors.

## Conclusion

In summary, the photoresponse of near infrared region photodetectors was studied with integration of monometallic of Au and Ag nanoparticles in a reduced graphene oxide. The formation of nanoparticles is based on fabrication of metallic thin layer using electron beam evaporation machine followed by solid-state dewetting approach. Under 680 nm illumination of light, our rGO photodetector exhibited the highest performance for Au-rGO with the highest responsivity of 67.46 AW^−1^ and the highest specific detectivity as 2.39 × 10^13^ Jones. Meanwhile, Ag-rGO achieved the highest responsivity of 17.23 AW^−1^, specific detectivity of 7.17 × 10^11^ Jones at 785 nm. The response time are 0.146 µs and 0.135 µs for Au-rGO and Ag-rGO respectively for both wavelength, which is an improvement results as compared to the previously reported NPs-rGO photodetectors. This prove that the presence of nanoparticles can manipulate strong-light matter interaction in graphene layer thus be a great advancing of NPs-rGO based detectors (Supplementary Information).

## Supplementary Information

Supplementary Information.
