# Supplementary material for: Plasmon-enhanced reduced graphene oxide photodetector with monometallic of Au and Ag nanoparticles at VIS–NIR region
Source: Sci Rep. 2021 Oct 4;11:19688. doi: 10.1038/s41598-021-99189-w (PMC8490468; doi:10.1038/s41598-021-99189-w)
Supplement: Supplementary file 1 — Supplementary Information. [file 41598_2021_99189_MOESM1_ESM.pdf]

APPENDIX

Table S1: Photoresponse measurement for rGO, Au-rGO and Ag-rGO at 680nm illumination light

Device	Optical power density, Popt (mW/cm2)	Photocurrent, Iph	Responsivity (A/W)	Detectivity (Jones)	EQE (%)
rGO	0.0083	0.000094	7.581	6.32E+11	1.38E+03
	0.0085	0.000192	15.092	1.26E+12	2.75E+03
	0.0097	0.000201	13.807	1.15E+12	2.52E+03
	0.0113	0.000220	12.970	1.08E+12	2.37E+03
	0.0169	0.000251	9.894	8.24E+11	1.80E+03
	0.0282	0.000343	8.118	6.76E+11	1.48E+03
	0.5568	0.000371	0.444	3.70E+10	8.09E+01
	1.1136	0.000371	0.222	1.85E+10	4.05E+01
Au	0.0083	0.000090	7.243	2.56E+11	1.32E+03
	0.0085	0.000484	38.018	1.35E+12	6.93E+03
	0.0097	0.000574	39.450	1.40E+12	7.19E+03
	0.0113	0.000716	42.262	1.50E+12	7.71E+03
	0.0169	0.001343	52.994	1.88E+12	9.66E+03
	0.0282	0.002853	67.463	2.39E+12	1.23E+04
	0.5568	0.003925	4.699	1.66E+11	8.57E+02
	1.1136	0.006261	3.748	1.33E+11	6.83E+02
Ag	0.0083	0.000149	11.953	4.98E+11	2.18E+03
	0.0085	0.000215	16.857	7.02E+11	3.07E+03
	0.0097	0.000266	18.293	7.61E+11	3.34E+03
	0.0113	0.000323	19.058	7.93E+11	3.48E+03
	0.0169	0.000678	26.729	1.11E+12	4.87E+03
	0.0282	0.000843	19.943	8.30E+11	3.64E+03
	0.5568	0.001039	1.244	5.18E+10	2.27E+02
	1.1136	0.001261	0.755	3.14E+10	1.38E+02
AuAg	0.0083	0.000015	1.207	1.31E+11	2.20E+02
	0.0085	0.000027	2.114	2.29E+11	3.86E+02
	0.0097	0.000041	2.832	3.07E+11	5.17E+02
	0.0113	0.000041	2.415	2.62E+11	4.40E+02
	0.0169	0.000045	1.771	1.92E+11	3.23E+02
	0.0282	0.000051	1.195	1.30E+11	2.18E+02
	0.5568	0.000051	0.061	6.62E+09	1.11E+01
	1.1136	0.000043	0.026	2.78E+09	4.67E+00

Table S2: Photoresponse measurement for rGO, Au-rGO and Ag-rGO at 785 nm illumination light

Device	Optical power density, Popt (mW/cm2)	Photocurrent, Iph	Responsivity (A/W)	Detectivity (Jones)	EQE (%)
rGO	0.03	0.000037	0.788	3.65E+10	1.24E+02
	0.10	0.000090	0.580	2.69E+10	9.16E+01
	4.49	0.000135	0.020	9.29E+08	3.17E+00
	6.54	0.000157	0.016	7.44E+08	2.53E+00
	8.96	0.000198	0.015	6.85E+08	2.33E+00
	11.48	0.000231	0.013	6.21E+08	2.12E+00
	13.00	0.000241	0.012	5.73E+08	1.95E+00
	15.08	0.000261	0.012	5.35E+08	1.82E+00
Au	0.03	0.000711	15.287	5.41E+11	2.41E+03
	0.10	0.000505	3.266	1.16E+11	5.16E+02
	4.49	0.000682	0.101	3.58E+09	1.60E+01
	6.54	0.000688	0.070	2.48E+09	1.11E+01
	8.96	0.000827	0.062	2.18E+09	9.72E+00
	11.48	0.001000	0.058	2.05E+09	9.17E+00
	13.00	0.001156	0.059	2.10E+09	9.37E+00
	15.08	0.001731	0.077	2.71E+09	1.21E+01
Ag	0.03	0.000801	17.233	7.17E+11	2.72E+03
	0.10	0.000801	5.182	2.16E+11	8.19E+02
	4.49	0.001054	0.156	6.51E+09	2.47E+01
	6.54	0.000940	0.096	3.99E+09	1.51E+01
	8.96	0.001007	0.075	3.12E+09	1.18E+01
	11.48	0.001159	0.067	2.80E+09	1.06E+01
	13.00	0.001449	0.074	3.09E+09	1.17E+01
	15.08	0.001709	0.076	3.15E+09	1.19E+01
AuAg	0.03	0.000105	2.255	2.45E+11	3.56E+02
	0.10	0.000116	0.747	8.11E+10	1.18E+02
	4.49	0.000694	0.103	1.12E+10	1.63E+01
	6.54	0.000956	0.098	1.06E+10	1.54E+01
	8.96	0.001210	0.090	9.77E+09	1.42E+01
	11.48	0.001457	0.085	9.18E+09	1.34E+01
	13.00	0.001691	0.087	9.41E+09	1.37E+01
	15.08	0.002042	0.090	9.80E+09	1.43E+01

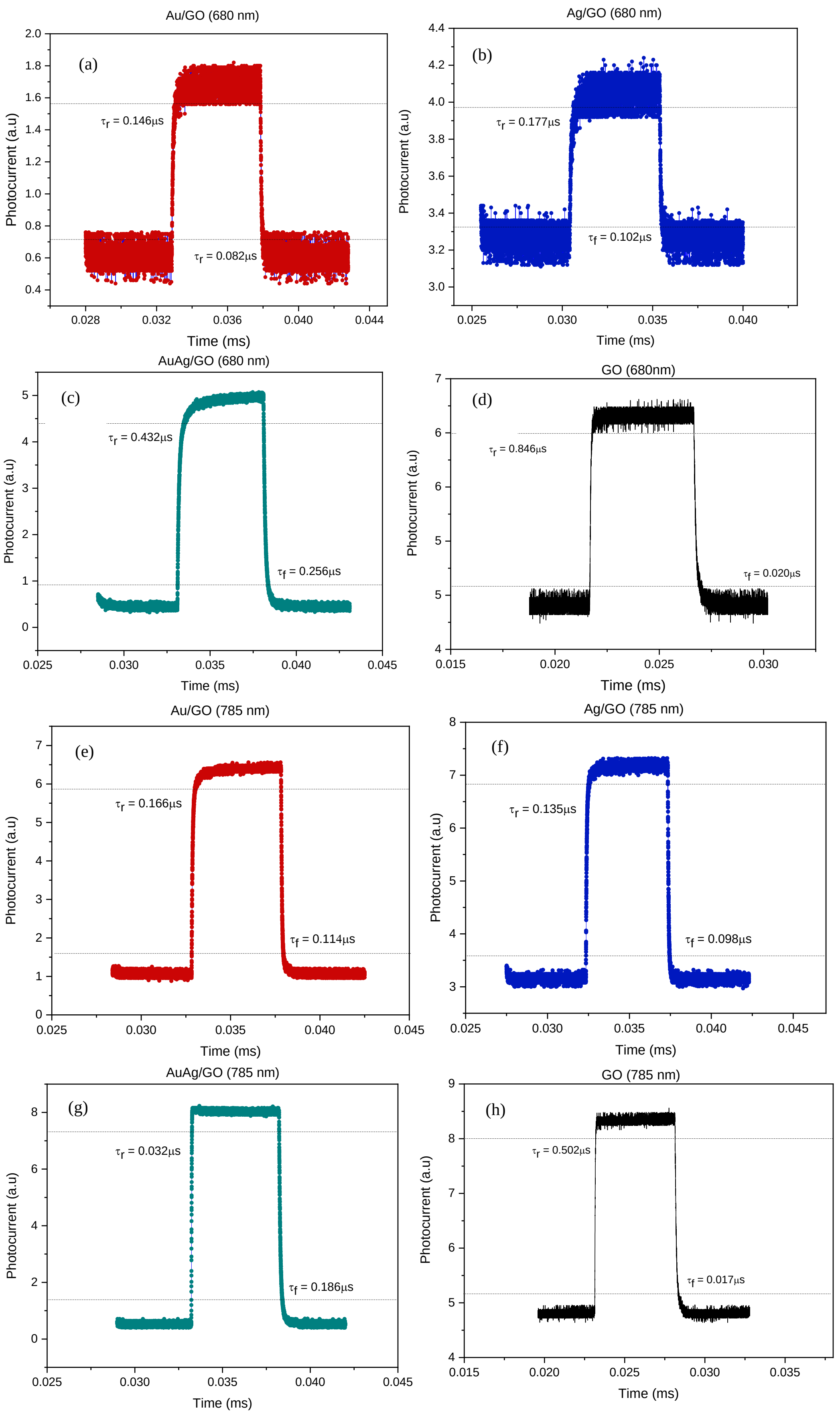

Figure S1: Rising and falling edges for estimating rise time (τ_r) and the fall time (τ_f) of all devices with (a,b,c,d) are under 680 nm laser illumination and (e,f,g.h) under 785 nm laser illumination.
